# Susceptibility to Ticks and Lyme Disease Spirochetes Is Not Affected in Mice Coinfected with Nematodes

**DOI:** 10.1128/IAI.01309-15

**Published:** 2016-04-22

**Authors:** Denny Maaz, Sebastian Rausch, Dania Richter, Jürgen Krücken, Anja A. Kühl, Janina Demeler, Julia Blümke, Franz-Rainer Matuschka, Georg von Samson-Himmelstjerna, Susanne Hartmann

**Affiliations:** a Institute of Immunology, Centre of Infection Medicine, Freie Universität Berlin, Berlin, Germany; b Environmental Systems Analysis, Institute of Geoecology, Technical University of Braunschweig, Braunschweig, Germany; c Institute of Parasitology and Tropical Veterinary Medicine, Freie Universität Berlin, Berlin, Germany; d Department of Medicine I for Gastroenterology, Infectious Disease, and Rheumatology, Research Center ImmunoSciences, Charité Berlin, Berlin, Germany; e Outpatient Clinic, University of Potsdam, Potsdam, Germany

## Abstract

Small rodents serve as reservoir hosts for tick-borne pathogens, such as the spirochetes causing Lyme disease. Whether natural coinfections with other macroparasites alter the success of tick feeding, antitick immunity, and the host's reservoir competence for tick-borne pathogens remains to be determined. In a parasitological survey of wild mice in Berlin, Germany, approximately 40% of Ixodes ricinus-infested animals simultaneously harbored a nematode of the genus Heligmosomoides. We therefore aimed to analyze the immunological impact of the nematode/tick coinfection as well as its effect on the tick-borne pathogen Borrelia afzelii. Hosts experimentally coinfected with Heligmosomoides polygyrus and larval/nymphal I. ricinus ticks developed substantially stronger systemic type 2 T helper cell (Th2) responses, on the basis of the levels of GATA-3 and interleukin-13 expression, than mice infected with a single pathogen. During repeated larval infestations, however, anti-tick Th2 reactivity and an observed partial immunity to tick feeding were unaffected by concurrent nematode infections. Importantly, the strong systemic Th2 immune response in coinfected mice did not affect susceptibility to tick-borne B. afzelii. An observed trend for decreased local and systemic Th1 reactivity against B. afzelii in coinfected mice did not result in a higher spirochete burden, nor did it facilitate bacterial dissemination or induce signs of immunopathology. Hence, this study indicates that strong systemic Th2 responses in nematode/tick-coinfected house mice do not affect the success of tick feeding and the control of the causative agent of Lyme disease.

## INTRODUCTION

Ticks are the most important arthropod vectors of pathogens in temperate regions of the Northern Hemisphere. In particular, ticks of the genus Ixodes transmit several pathogens affecting the health of humans and livestock, such as Borrelia spp., which cause Lyme disease (LD) or relapsing fever, and Rickettsia spp. of the spotted fever group, which cause diseases such as aneruptive fever and tick-borne lymphadenopathy (1). Small rodents serve as reservoir hosts for many tick-borne pathogens, and the larval stage of Ixodes ricinus, the main vector species in Western and Central Europe, frequently feeds on mice and voles (2). The larval tick stage efficiently acquires the pathogen when feeding on infected rodents (3). After they molt, nymphal ticks constitute the major source for infections in people (4). Two genospecies of LD spirochetes account for the majority of infections in Eurasia, Borrelia garinii and Borrelia afzelii, the latter of which mainly and efficiently perpetuates in rodents (5–8). Mice and voles in urban and rural areas are frequently infected by intestinal helminths, such as the trichostrongyloid Heligmosomoides polygyrus (9–11), raising the question of whether such natural coinfections may affect the feeding success of ticks and the transmission of tick-borne pathogens.

Ticks initiate type 2 T helper cell (Th2) responses during their blood meal (12). Although feeding of I. ricinus ticks results in protective immunity in guinea pigs and rabbits, animals nonnative to this tick's distribution, the immune responses of most autochthonous wild mouse species and laboratory mouse strains are insufficient to protect them from repeated infestations with ticks (12–16). On the other hand, feeding Ixodes ticks suppress Th1 responses against tick-borne LD spirochetes in mice (17), and the suppression of Th2 cytokines or the reconstitution of tumor necrosis factor alpha, interleukin-2 (IL-2), and gamma interferon (IFN-γ) after tick attachment reduces infection rates in disease-susceptible laboratory mouse strains (18, 19). Infections with the enteric nematode H. polygyrus elicit strong local and systemic Th2 and regulatory responses (20, 21) and thereby affect the Th1-dependent control of concomitant bacterial and protozoan infections (22–28). It may be that the concurrent presence of enteric nematodes synergistically enhances Th2 and associated innate effector cell responses toward repeated tick infestations and as a result affects tick feeding success and the transmission of tick-borne pathogens. The expected strong general skewing of immune responses toward a Th2 response in mice simultaneously harboring the two macroparasites H. polygyrus and I. ricinus may restrain the immune control of tick-borne pathogens and subsequently increase the reservoir competence of rodents.

To investigate how frequently mice simultaneously harbor enteric nematodes and ticks in nature, we captured wild rodents in Berlin, Germany, and determined the frequency of infection with Heligmosomoides spp. and infestation with I. ricinus. Using a defined experimental laboratory system, we then assessed parameters of antitick immunity and the competence of the mice as a reservoir for LD spirochetes using inbred house mice. To examine whether the expected strong Th2 immune responses associated with intestinal nematode infections interfere with, first, the experimental host's development of antitick immunity (measured by means of determination of tick-specific cytokine responses and the success of repeated tick feeding) and, second, the control of tick-borne pathogens, we established an experimental model of coinfection in inbred laboratory mice, compared susceptibility to tick feeding and to tick-borne infection with LD spirochetes, and evaluated local and systemic immune responses.

Here we show that (i) H. polygyrus and I. ricinus frequently parasitize wild Apodemus mice in Berlin; (ii) local anti-I. ricinus immune responses in laboratory house mice are not altered by a concurrent nematode infection, despite the induction of extraordinarily high systemic Th2 responses by nematode/tick coinfections; (iii) the nematode infection does not alter the feeding success of pathogen-free tick larvae and spirochete-infected nymphs or the development of partial immunity toward repeated tick infestations; and (iv) the nematode coinfection does not affect the tick-borne transmission, replication, and dissemination of B. afzelii spirochetes in house mice.

## MATERIALS AND METHODS

### Wild rodent trapping.

Wild rodents were trapped at two urban study sites (Moabit, Steglitz) and two periurban study sites (Gatow, Tegel) in Berlin, Germany, in November 2010 and from April to November 2011 (license number G0210/10, Landesamt für Gesundheit und Soziales, Berlin, Germany). The sites, trapping method, and type of euthanasia used were described by J. Krücken, J. Blümke, D. Maaz, J. Demeler, S. Ramünke, D. Antolová, R. Schaper, and G. von Samson-Himmelstjerna (submitted for publication). After the animals were transferred to the laboratory, the fur was screened for ticks under a stereomicroscope. Subsequently, a complete necropsy was performed, the gastrointestinal tract was thoroughly screened for Heligmosomoides spp., and any Heligmosomoides organisms detected were stored in 80% ethanol. The remaining carcass was placed on metal grids over water in screw-top jars to allow detachment of the remaining ticks. The water and the rodent body were examined during the following week. Ticks were stored in 70% ethanol. The parasite species were determined microscopically according to the morphological literature for nematodes (29) and ticks (30–32).

### Laboratory mice, parasites, and Borrelia.

Six- to 8-week-old specific-pathogen-free (SPF) female C57BL/6JRj, BALB/cJRj, and C3H/HeNRj mice (Janvier, France) were used for the experiments. The trichostrongyloid nematode H. polygyrus was maintained by serial passage in C57BL/6 mice. Specific-pathogen-free larval I. ricinus ticks were obtained from batches of eggs from two adult females after they had fed on laboratory beagle dogs (license number H0078/10, Landesamt für Gesundheit und Soziales, Berlin, Germany). The absence of tick-borne pathogens in the tick larvae used for experimentation was surveyed by published PCRs (33, 34) for the detection of a 153-bp fragment of the *hbb* gene of Borrelia burgdorferi sensu lato, a 203-bp fragment of the *gltA* gene of spotted fever Rickettsia spp., a 602- to 639-bp fragment of the 18S rRNA gene of piroplasmida (33), and a 257-bp fragment of the 16S rRNA gene of the Anaplasmataceae (34) in DNA isolated (33) from subsets of 20 larvae randomly chosen from each tick batch. Borrelia afzelii-infected I. ricinus nymphs were derived from larvae that had engorged on experimentally infected mice (license number 23-2347-A-24-1-2010, Landesamt für Umwelt, Gesundheit, und Verbraucherschutz, Potsdam, Germany).

### Animal experimentation.

Mice were infected with 250 H. polygyrus L3 larvae via oral gavage. In an initial experiment, mice of three strains (C57BL/6, BALB/c, and C3H) were infested with I. ricinus larvae on day 15 after H. polygyrus infection and dissected 20 days later. To survey the feeding success of larval ticks and the antilarval tick immune responses, C57BL/6 mice were infected with H. polygyrus and subsequently repeatedly infested with 50 specific-pathogen-free I. ricinus larvae by biweekly tick infestations on days 4, 18, and 34 postinfection (p.i.) in order to cover the early phase of local Th2 reactivity (at approximately day 6 p.i.), the phase of maximal Th2 reactivity (at about day 21 p.i.), and the chronic phase of infection with a declining magnitude of the nematode-induced Th2 reaction (at approximately day 35 p.i.). Mice exposed either to nematodes or to ticks and naive mice served as controls. To investigate the effect of H. polygyrus on the control of a tick-borne pathogen, nematode-infected and -free groups were infested with 4 to 6 B. afzelii-infected I. ricinus nymphs on day 16 after H. polygyrus infection in order to challenge the mice with B. afzelii at the peak of nematode-induced Th2 reactivity. Age-matched naive mice served as controls. During the period of tick infestation, all mice, including the naive controls, wore a collar to prevent removal of the ticks placed in the head region. Mice were housed individually at 80% humidity in filter-topped cages in ventilated cabinets. To optimize the recovery of detached ticks, the mice were kept on metal grids over water, and food was restricted to 3 g per day during tick feeding. Mice received water *ad libitum*. In several experiments, mice were anesthetized with isoflurane once a week for retroorbital blood collection with hematocrit capillary tubes. To assess the feeding success of the ticks, I. ricinus larvae or nymphs detaching from hosts were collected from the water and counted twice a day, pooled for every mouse in a screen-capped glass vial, and transferred to a desiccator with supersaturated MgSO_4_ solution. The feeding duration was calculated as the mean recovery time of engorged ticks. Seven days after all ticks had detached, the engorgement weight of the pooled ticks was determined using a microscale, and the mean weight was calculated. The molting rates of fed larvae from the 1st and 2nd infestations or of fed nymphs were determined 3 months after infestation. All animal experiments were approved by and conducted in accordance with the guidelines of the appropriate committee (Landesamt für Gesundheit und Soziales, Berlin, Germany) under license number G0282/12.

### Preparation of nematode, tick, and spirochete Ags.

H. polygyrus antigen (Ag) was prepared as described previously (28). Antigen was acquired from specific-pathogen-free feeding I. ricinus larvae 48 h after attachment to C57BL/6 mice at a time of intensive saliva production. The ticks were removed from the host, washed in sterile phosphate-buffered saline (PBS), homogenized, and sonicated (3 times for 10 s each time, 60 W) in PBS on ice, and the sonicate was centrifuged at 20,000 × *g* for 10 min at 4°C. The supernatant was sterile filtered through 0.22-μm-pore-size filters (Millipore, USA). To acquire B. afzelii antigen, spirochetes cultured in Barbour-Stoenner-Kelly H medium (Sigma-Aldrich, Germany) supplemented with 6% rabbit serum were washed three times by centrifugation at 10,000 × *g* and 4°C for 30 min each time in PBS containing 5 mM MgCl_2_ and sonicated on ice (8 times for 15 s each time), and the sonicate was centrifuged at 10,000 × *g* at 4°C for 30 min. The protein contents of the supernatants were determined by use of a bicinchoninic acid protein assay kit (Pierce, USA). The antigens were stored at −80°C until use.

### Cell culture.

Splenocytes and cells were isolated from the gut-draining and head-skin-draining lymph nodes of euthanized mice by passing the organs through a 70-μm-mesh-size cell strainer (BD, USA). The cells were cultured as triplicates of 3.5 × 10^5^ to 5 × 10^5^ cells in 200 μl RPMI 1640 medium (PanBiotech, Germany) containing 10% fetal calf serum, 20 mM l-glutamine, 100 U/ml penicillin, and 100 μg/ml streptomycin. The cultures were stimulated with 40 μg/ml worm or tick Ag, 30 μg/ml B. afzelii Ag (all for 72 h), or 1 μg/ml anti-CD3 and anti-CD28 (BD, USA) for 48 h at 37°C in a 5% CO_2_ atmosphere. The supernatants were stored at −20°C for cytokine detection.

### Immunohistology.

Formalin-fixed tissue samples were dehydrated and embedded in paraffin. Tibiotarsal joints were decalcified before they were embedded in paraffin. The tissues in the paraffin sections (1 to 2 μm) were dewaxed, hydrated, and histochemically stained with hematoxylin and eosin (Merck) to obtain an overview, toluidine blue to observe mast cells, and a modified Sirius red staining protocol to observe eosinophil granulocytes (35). Sections were stained with toluidine blue (Sigma) for 10 min, washed with distilled water, and dehydrated by dipping them in 1% acetic acid and 100% ethanol. Sections were cleared in xylol, and coverslips were mounted with Corbit balsam (Hecht). For staining of eosinophils, sections were stained for 1 min with hematoxylin (Merck), washed with tap water, and dehydrated with 100% ethanol before staining with direct red 80 dye (Sigma) for 2 h. Sections were washed with tap water, dehydrated with 100% ethanol, and cleared in xylol before coverslips were mounted with Corbit balsam. Images were acquired using a fluorescence microscope (AxioImager Z1) equipped with a charge-coupled-device camera (AxioCam MRm) and processed with Axiovision software (Carl Zeiss AG, Germany).

### ELISA.

Culture supernatants were screened by enzyme-linked immunosorbent assay (ELISA) for IFN-γ, IL-13 (Ready-Set-Go! ELISA; eBioscience, USA), and IL-10 (BD, USA) according to the manufacturer's instructions.

### Flow cytometry.

Blood samples were placed in PBS with 0.2% bovine serum albumin (BSA) and 5 mM EDTA, and cells were stained with anti-CD4–fluorescein isothiocyanate (clone RM4-5; eBioscience, USA) and fixable viability dye eFluor 780 (eBioscience, USA) for dead cell exclusion. Red blood cells were lysed in fluorescence-activated cell sorting (FACS) lysing solution (BD, USA), before leukocytes were stained intracellularly with anti-Foxp3–eFluor 450 (clone FJK-16S; eBioscience, USA), anti-GATA-3–eFluor 660 (clone TWAJ; eBioscience, USA), and anti-T-bet–phycoerythrin (PE) (clone eBio4B10; eBioscience, USA) using a fixation/permeabilization buffer kit (eBioscience, USA). Cells from the spleen and lymph nodes were labeled with anti-CD4–peridinin chlorophyll protein–eFluor 710 (clone GK1.5; eBioscience, USA) and fixable viability dye eFluor 780 (eBioscience, USA) in PBS with 0.2% BSA. Transcription factors were stained as described above for the white blood cells. For the intracellular detection of cytokines, cells were incubated with 1 μg/ml phorbol myristate acetate (PMA) and ionomycin for 30 min and 5 μg/ml brefeldin A (Sigma, Germany) for another 2.5 h. Subsequently, they were stained with anti-IFN-γ–eFluor 450 (clone XMG1.2; eBioscience, USA), anti-IL-13–Alexa Fluor 647 (clone eBio13A; eBioscience, USA), anti-IL-10–PE (clone JES5-16E3; eBioscience, USA), and anti-IL-17–Alexa Fluor 488 (clone TC11-18H10; BD, USA). Cells were acquired using LSRII and Canto II flow cytometers (BD, USA) and analyzed using FlowJo (version 8.8.7) software (TreeStar, USA).

### Spirochete quantification.

For quantification of spirochetes in mouse organs in relation to tissue weight, a touchdown quantitative PCR (qPCR) assay for absolute quantification was developed, and λ phage genome copies were used as an external standard. For DNA isolation, 80 ± 1 mg of head skin and 70 ± 1 mg of heart were sampled with sterile scalpel blades, and the skinned left tibiotarsal joint (weight range, 32.6 to 39.1 mg) was obtained using clean scissors. Prior to DNA isolation with a FastDNA Spin kit with Lysing Matrix A (MP Biomedicals, France), 10^4^ copies of the 48,502-bp λ phage genome (Thermo Fisher Scientific, USA) per mg head skin or heart and 10^5^ copies per tibiotarsal joint were added as an external standard for tissue weight. To calculate copy numbers, the DNA concentration (0.3 μg/μl, measured spectrophotometrically) and the molecular weight of the genome were used. Samples were homogenized with a FastPrep 24 kit (MP Biomedicals, France) 4 times for 30 s each time at 6.0 m/s and cooled on ice between homogenization steps. Variations in the amount of DNA in the samples used in the qPCR have a major impact on the accuracy of absolute quantification, since qPCR efficiency is decreased with higher DNA concentrations. As common spectrophotometric DNA quantification is strongly impacted by contamination with salts and proteins, a more precise fluoroscopic DNA quantification assay for fluorescence measurement was developed using LCGreen Plus 10× dye (BioChem, USA), exclusive binding on double-stranded DNA (dsDNA), and a LightCycler 480 II system (Roche Molecular Diagnostics, USA). Accurate DNA quantification over the range of 1 to 100 ng was obtained. DNA samples, standards (20, 40, 60, 80, and 100 ng λ phage DNA), and water controls were prepared in duplicate in a 10-μl assay volume for each well in 96-well plates. After vigorous vortexing of the sealed plates, samples were centrifuged and fluorescence was measured by the LightCycler 480 II system after 2 min of incubation at 25°C during a short heating from 25 to 26°C with 100 acquisitions per degree Celsius. For extrapolation of the dsDNA quantity in the samples, a calibration curve was calculated from the fluorescence values of the standards using a 4-parameter logistic regression model. Two real-time PCR assays targeting a 153-bp fragment of the *hbb* gene of LD spirochetes and a 158-bp fragment of the λ phage genome were conducted with the LightCycler 480 II system using LightCycler 480 SYBR green I Master mix (Roche Molecular Diagnostics, USA), 0.5 μM each primer, and exactly 100 ng sample DNA in a 20-μl volume. The primers used for the *hbb* fragment are published elsewhere (36), and the forward and reverse primers used for the λ phage genome were 5′-TTTTGATTTTGCTGTTTCAA-3′ and 5′-ACCTTTTCCATGAATTGGTA-3′, respectively. All PCRs were performed in duplicate and included a negative control and standards, each with 100 ng target-free mouse DNA from the respective organ. The standards consisted of a dilution series of 10^4^ to 10^1^ and 3 copies of a plasmid carrying the *hbb* gene standard or λ phage DNA, respectively. The thermocycling conditions consisted of an initial denaturation step at 95°C for 5 min, followed by 50 cycles of 95°C for 5 s, annealing for 30 s (at an initial temperature of 62°C and then a decrease in the temperature by 0.25°C/cycle to 57°C), and 72°C for 15 s. Touchdown amplification was performed to increase the binding specificity of the primers during the first cycles. After amplification, a high-resolution melting step consisting of 95°C for 5 s, 40°C for 1 min, and progressive heating from 65 to 95°C with 20 acquisitions per degree Celsius was performed to identify target fragments by melting temperature in combination with their fragment size in subsequent agarose gel electrophoresis. The numbers of Borrelia *hbb* copies extrapolated from the standard curve were multiplied by the ratio of the extrapolated λ phage DNA copy numbers in the sample and the number of copies previously added per milligram of skin/heart or per tibiotarsal joint. The detection limit was less than 3 copies, and the quantification limit was 5 to 10 copies per 100 ng DNA for both touchdown qPCR assays.

### Statistics.

The confidence intervals (CIs) of the prevalences were calculated as Wilson score intervals using R Statistics software and the PlotCIs package. Negative binomial regression of tick numbers was performed using R Statistics software and the MASS package to estimate the influence of the variable Heligmosomoides sp. count and the categorical variables mouse species (Apodemus flavicollis and Apodemus sylvaticus) and trapping location (Gatow, Moabit, Steglitz, and Tegel) on the number of I. ricinus ticks on wild mice.

GraphPad Prism (version 5.01) software (San Diego, CA, USA) was used for the plotting and statistical analysis of the experimental infection data. The data were tested for statistically significant differences using an unpaired *t* test, one-way analysis of variance (ANOVA) with a Bonferroni-corrected posttest (see Fig. 3 and 5), and two-way-ANOVA (see Fig. 2) under the assumption of a normal distribution of the FACS fluorescence data, ELISA optical density data, and the majority of the experimental parasite infection data. The posttests following the two-way ANOVA for individual pairs using the Bonferroni correction were performed with the posttest calculator (GraphPad Software). The number of engorged tick nymphs and the molting rate in Fig. 7A and D, respectively, and the cell counts in Fig. S1B in the supplemental material were not normally distributed, and differences were tested using the Mann-Whitney U test. Differences with *P* values below 0.05 were considered to be significant.

## RESULTS

### Nematode/tick coinfection is frequent in urban and periurban wild mice.

**FIG 1:**
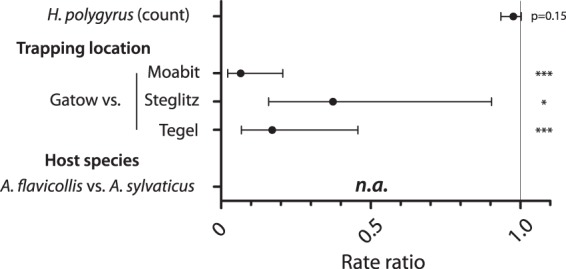
Incident rate ratios with 95% CIs for risk factors affecting the number of host-associated Ixodes ricinus ticks on wild mice. A negative binomial regression for the I. ricinus count (larvae and nymphs) as the dependent variable was performed using the H. polygyrus worm count and the categorical factors trapping location with three dummy variables and host species as independent variables. Reference categories for the trapping location and host species were Gatow and A. flavicollis, respectively. *, *P* < 0.05; ***, *P* < 0.001; n.a., not applicable because of total colinearity with the trapping location. Data are for 107 wild mice.

A total of 107 potential murine hosts of a Heligmosomoides sp. representing 82 yellow-necked mice (Apodemus flavicollis) and 25 long-tailed wood mice (A. sylvaticus) were trapped alive at 2 urban sites (Moabit, Steglitz) and 2 periurban sites (Gatow, Tegel) in Berlin and examined for their parasite burden. No house mice (Mus musculus) were caught, as the traps were placed exclusively outside buildings. Both species frequently hosted H. polygyrus (47.7%; 95% CI, 38.5 to 57.0%; *n* = 107) with intensities of from 1 to 246 specimens (Table 1). In addition, 55.1% (95% CI, 45.7 to 64.2%) were infested by up to 59 subadult I. ricinus ticks (92.1% larvae, 7.9% nymphs). A high percentage (41.3%; 95% CI, 29.1 to 53.4%) of tick-infested mice (*n* = 59) were simultaneously infected by H. polygyrus. A negative binomial regression model including all 107 mice showed an important influence of the trapping location and/or the rodent species on the number of I. ricinus ticks on the rodents but could not distinguish between the two variables because of total colinearity (Fig. 1). A. sylvaticus mice were exclusively trapped in Moabit, whereas A. flavicollis occurred only at the three other locations. A. flavicollis mice from Gatow were infested with significantly more ticks than those from Steglitz (*P* < 0.05) and Tegel (*P* < 0.001) and A. sylvaticus mice from Moabit (*P* < 0.001). Although the difference was not significant (*P* = 0.15), the number of Heligmosomoides organisms showed a negative trend versus the number of ticks, with every nematode reducing the number of ticks by 2.4% (95% CI, −6.6% [decrease] to +0.17% [increase]). Likelihood ratio tests confirmed that the negative binomial model was more appropriate than a Poisson model (*P* < 0.001) and a significant improvement over the null model without coefficients (*P* < 0.001). In order to assess a possible influence of a nematode infection on the parameters of tick infestation, host development of antitick immunity, and dissemination of tick-borne pathogens, we established an experimental nematode/tick coinfection model under defined laboratory conditions with house mice.

**TABLE 1:** Numbers of wild Apodemus (Sylvaemus) mouse species trapped at the different study sites and I. ricinus and H. polygyrus infection parameters^*a*^

Trapping location			Ixodes ricinus (larvae and nymphs) infection parameters				*H*. polygyrus infection parameters			
	No. of mice trapped		Prevalence		Intensity		Prevalence		Intensity	
	Apodemus flavicollis	Apodemus sylvaticus	% of specimens	95% CI	Mean	Range	% of specimens	95% CI	Mean	Range
Gatow	33		81.8	65.6–91.4	15.7	1–52	9.1	3.1–23.6	2.7	1–6
Tegel	22		54.5	34.7–73.1	3.8	1–9	40.9	23.3–61.3	24.1	1–200
Steglitz	27		51.9	34.0–69.3	8.9	1–59	66.7	47.8–81.4	7.6	1–52
Moabit		25	24.0	11.5–43.4	2.5	1–6	84.0	65.4–93.6	29.2	1–246
Total^*b*^	82	25	55.1	45.7–64.2	10.3	1–59	47.7	38.5–57.0	19.1	1–246

a Prevalences (in percent with 95% CIs) and intensities (mean number of parasites per infected/infested mouse and range) are shown for H. polygyrus and subadult live stages of I. ricinus.

b Total, the sums of the number of trapped mice per species as well as the infection parameters for all trapped mice (*n* = 107).

### Nematode/tick coinfection leads to highly polarized Th2 responses in inbred mouse strains.

**FIG 2:**
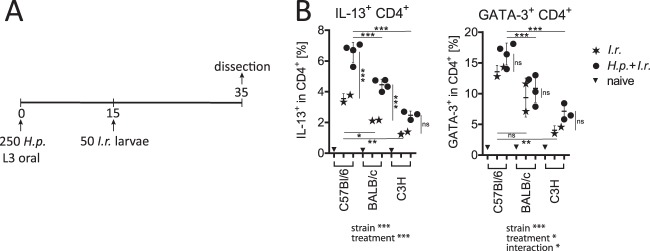
Comparison of systemic Th2 responses to tick and nematode/tick coinfections in three inbred mouse strains. (A) Experimental setup showing the time line (in days) of exposure to H. polygyrus (*H.p.*) and to I. ricinus (*I.r.*) larvae. (B) Frequencies (mean ± SD) of splenic IL-13^+^ CD4^+^ T cells in response to PMA-ionomycin stimulation (left) and GATA-3^+^ CD4^+^ cells (right) detected by flow cytometry. Values of significance determined by two-way ANOVA are shown below the graph. Values of significance determined by individual Bonferroni-corrected pairwise posttests are depicted with horizontal lines for comparisons between mouse strains (lines at the top are for mice coinfected with H. polygyrus and I. ricinus; lines at the bottom are for mice infested with I. ricinus) and vertical lines for comparison between treatments. *, *P* < 0.05; **, *P* < 0.01; ***, *P* < 0.001; ns, not significant. Data are for 1 to 4 mice per group.

To compare the spectrum of Th2 reactivity against ticks and nematodes depending on the host's genetic background, mice of three inbred strains with reported differences in immune reactivity (13, 37, 38) were either infested with I. ricinus larvae alone or infested with I. ricinus larvae and coinfected with the nematode H. polygyrus (Fig. 2A). Systemic Th2 responses in spleens were characterized by flow cytometry 20 days after I. ricinus infestation on the basis of cytokine (IL-13) production and expression of the lineage transcription factor of Th2 cells, GATA-3 (Fig. 2B). Coinfected mice of all strains exhibited elevated Th2 responses compared to the responses of the controls infested only with ticks. C57BL/6 mice produced more splenic IL-13-producing (IL-13^+^) CD4^+^ cells and exhibited higher frequencies of GATA-3-expressing (GATA-3^+^) CD4^+^ cells in response to coinfection or infestation with ticks only than BALB/c and C3H mice. Following the hypothesis that a strong Th2 response during nematode/tick coinfection might affect the feeding success of ticks and the control of tick-borne pathogens, C57BL/6 mice were chosen for subsequent experiments.

### The frequencies of Th2 cells peak during acute nematode and tick coinfection.

**FIG 3:**
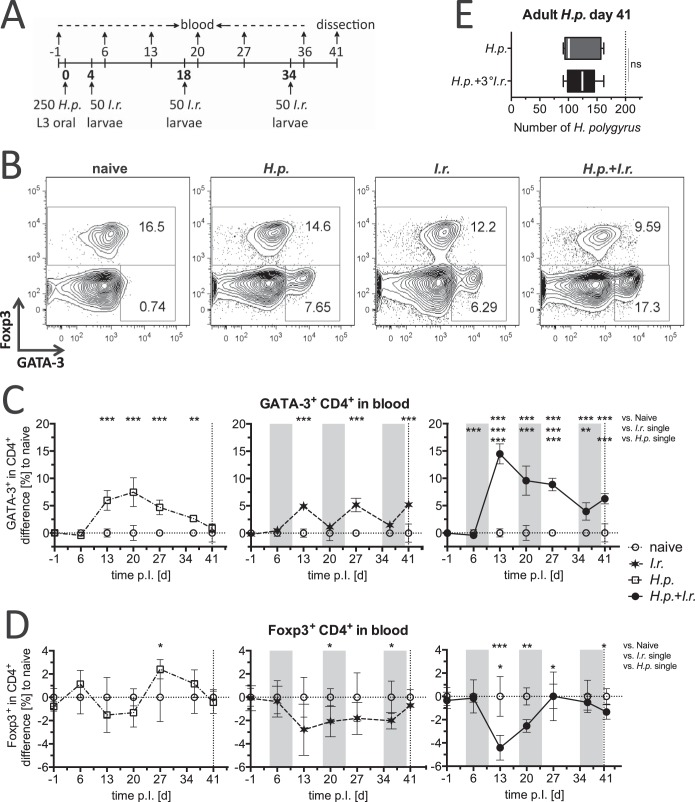
Kinetics of GATA-3^+^ CD4^+^ Th2 cells from C57BL/6 mice during infestation with I. ricinus ticks alone and during infestation with I. ricinus ticks and coinfection with H. polygyrus. (A) Experimental setup showing the time line (in days) of exposure to H. polygyrus (*H.p.*) and to I. ricinus (*I.r.*) larvae and blood collection. (B) Representative flow cytometry density plots of peripheral blood CD4^+^ cells stained for Foxp3 and GATA-3 on day 13. (C, D) Frequencies of GATA-3^+^ (C) and Foxp3^+^ (D) CD4^+^ cells (mean ± SD) in the peripheral blood of H. polygyrus-infected mice (left), I. ricinus-infested mice (middle), and H. polygyrus- and I. ricinus-coinfected mice (right) normalized to those in the peripheral blood of naive controls. Gray shading and vertical lines, period of larval tick feeding and the day of dissection, respectively. One-way ANOVAs with a Bonferroni-corrected pairwise *t* test were performed for every time point in the kinetic analysis. d, day. (E) Number of adult H. polygyrus worms isolated from small intestines on day 41. The box plot shows the medians and quartiles, with whiskers indicating 95% CIs. Differences were tested using an unpaired *t* test. *, *P* < 0.05; **, *P* < 0.01; ***, *P* < 0.001; ns, not significant. Data are for 5 or 6 mice per group.

To evaluate the effects of a nematode infection on antitick immune responses, we next determined the kinetics of the systemic Th2 and regulatory T cell responses provoked by the nematode infection, repeated tick infestations, and a coinfection regimen with repeated tick feeding. We chose an infective dose of 250 H. polygyrus L3-stage larvae and 50 I. ricinus larvae to reflect the parasite burdens also detectable in wild mice (Table 1). C57BL/6 mice were either infested with specific-pathogen-free I. ricinus larvae three times at biweekly intervals or first infected with H. polygyrus and subsequently infested three times with I. ricinus starting on day 4 after nematode infection (Fig. 3A), a regimen chosen to cover the early, acute, and chronic phases of nematode infection reflecting expanding, maximal, and declining nematode-driven Th2 reactivity, respectively. Mice infected only with H. polygyrus and naive littermates served as controls. To survey the development of systemic Th2 and regulatory responses, blood was sampled once a week and GATA-3 and Foxp3 protein expression by CD4^+^ T cells was monitored (Fig. 3B). Infection with H. polygyrus alone led to the typical pattern of a strong systemic Th2 response during the acute phase of infection and the subsequent decline of Th2 cell levels to near the background levels in the chronic phase of infection (Fig. 3C), despite the presence of high worm burdens at the end of the experiment on day 41 p.i. (Fig. 3E). Tick infestations resulted in strong systemic Th2 responses of similar magnitudes after each infestation and a subsequent decline in Th2 cell frequencies (Fig. 3C). In mice coinfected with both parasites, systemic Th2 cells were substantially more numerous, roughly reflecting the sum of the Th2 cell populations detected in controls infected/infested with a single pathogen. Interestingly, the suppression of nematode-elicited systemic immune responses in the chronic phase of H. polygyrus infection at day 41 did not spill over to tick-induced responses, as GATA-3 cells expanded systemically in response to the third tick infestation with a magnitude similar to that in the controls infested with ticks only (Fig. 3C). Infections with H. polygyrus activate Foxp3-expressing regulatory T cells (Tregs), which control the developing Th2 response (39–41). The frequencies of Foxp3^+^ Tregs in the peripheral blood of mice exposed only to H. polygyrus or only to I. ricinus were not significantly altered compared to those in the peripheral blood of the naive controls (Fig. 3D). In coinfected mice, however, the dominant increase in GATA-3-expressing Th2 cells led to a reciprocal transient decline in the frequencies of Tregs detected in peripheral blood. Hence, we found that systemic Th2 responses were greatly increased in mice that were acutely infected by intestinal nematodes and concomitantly infested by ticks.

### Th2 cytokine responses to ticks are not affected by concurrent nematode infection.

**FIG 4:**
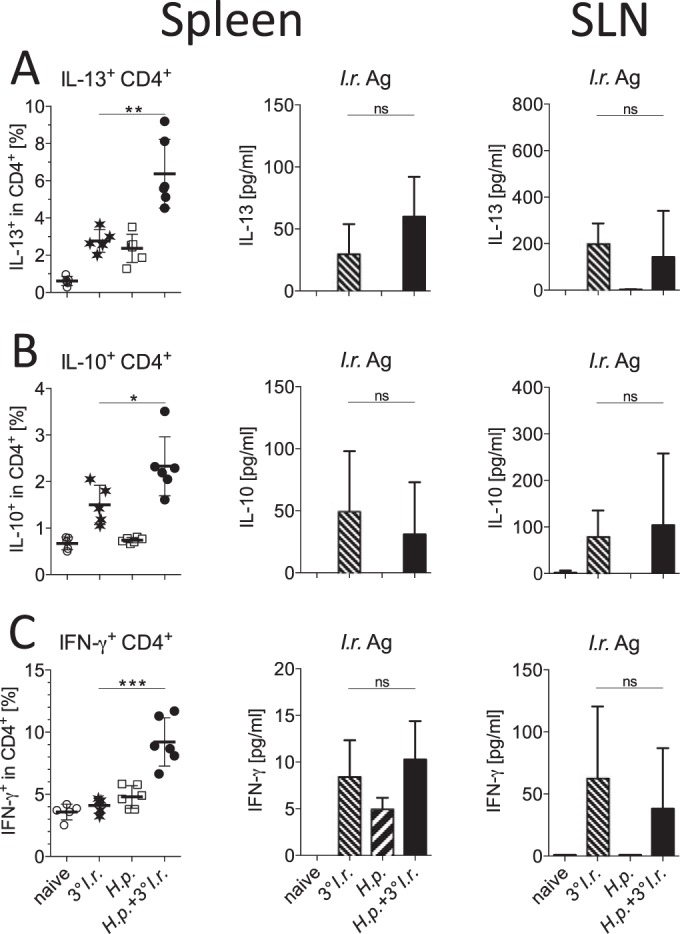
Systemic and local cytokine responses of mice infected with H. polygyrus and I. ricinus. The production of IL-13 (A), IL-10 (B), and IFN-γ (C) was measured in spleen CD4^+^ T cells by flow cytometry after intracellular cytokine staining following PMA-ionomycin stimulation (left) and in bulk culture supernatants of spleen and SLN cells by ELISA after specific stimulation with larval I. ricinus antigen (middle and right, respectively). Means and SDs are shown. Symbols represent the intracellular cytokine responses detected in cells from individual mice. Differences between groups were tested using an unpaired *t* test. *, *P* < 0.05; ** *P* < 0.01; ***, *P* < 0.001; ns, not significant. Data are for 5 or 6 mice per group.

We next analyzed whether systemic and local cytokine responses to repeated tick infestations were affected by a concurrent nematode infection. For this purpose, mice were dissected 1 day after the last I. ricinus larva of the third infestation had detached, and cells from spleen and local skin-draining lymph nodes (SLNs) were harvested and stimulated for cytokine production. As expected, the frequencies of splenic CD4^+^ T cells producing IL-13 in response to unspecific PMA-ionomycin-induced stimulation were the highest in coinfected mice (Fig. 4A). In response to larval tick antigen, however, similar levels of IL-13 were detected in cultures of splenocytes from coinfected mice and control mice infested only with ticks, a pattern also reflected by anti-CD3/anti-CD28 antibody-induced stimulation (Fig. 4A and not shown). The tick-specific local IL-13 responses of cells from cervical and axillary lymph nodes draining the head and neck skin region were also equivalent (Fig. 4A). A similar pattern was detected for IL-10: the highest frequencies of IL-10^+^ CD4^+^ cells were detected after unspecific activation of spleen cells from coinfected/infested mice, and systemic and local I. ricinus-specific IL-10 responses were equal in both tick-infested groups (Fig. 4B). Splenocytes from coinfected mice contained the highest proportion of CD4^+^ cells responding to PMA-ionomycin with IFN-γ production; however, IFN-γ-positive (IFN-γ^+^) responses to tick antigen in spleen cells were close to the detection limit of the assay and similar in spleen and SLNs for both tick-infested groups (Fig. 4C). Because Th2-associated innate effector cells contribute to immunity against ticks (42, 43), we compared the mast cell and eosinophil numbers of tick-infested and H. polygyrus-infected mice by histological analyses of the skin region affected by repeated tick feeding. They did not vary between the tick-infested experimental groups (see Fig. S1 in the supplemental material). Taken together, mice coinfected with nematodes and ticks develop very strong systemic Th2 responses, while local antitick immune responses are unaltered by an infection with enteric nematodes.

### Tick feeding success is not affected by concurrent H. polygyrus infection.

**FIG 5:**
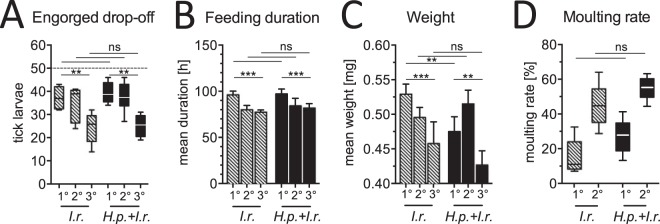
Feeding success of I. ricinus larvae on mice with and without H. polygyrus coinfection. (A and D) The number of engorged larvae recovered after they dropped off the mice (A) and the rates of molting to the nymph stage by engorged larvae (D) are depicted as box plots (medians and quartiles, with whiskers representing 95% CIs). (B and C) The feeding duration (B) and tick weight after detachment (C) are shown as means ± SDs. The dotted line in panel A depicts the total number of ticks applied to individual mice. Asterisks indicate the significance values obtained by Bonferroni-corrected pairwise posttests following one-way ANOVA. *, *P* < 0.05; **, *P* < 0.01; *** *P* < 0.001; ns, not significant. Data are for 5 or 6 mice per group.

In line with the unaltered tick-specific local cytokine responses in coinfected mice, the parameters of the success of tick feeding and subsequent molting were unaffected by the presence of H. polygyrus (Fig. 5). During successive infestations, the number of successfully engorged larval ticks and their feeding duration did not differ among the single infection and coinfection groups. Although ticks feeding on nematode-infected hosts during the 1st infestation were significantly lighter than ticks feeding on nematode-free mice, no such difference was observed during subsequent infestations. The effect of partial immunity against larval ticks after repeated feeding was also similar in both groups when the detachment rates of engorged ticks of the primary and tertiary infestations, their feeding durations, and their weights were compared (Fig. 5). The rates of molting to the nymphal stage did not differ for ticks feeding on worm-free and worm-infected hosts after the primary and secondary infestations. Ticks from the tertiary infestations, unfortunately, succumbed to fungal growth. Hence, we detected a partial protection of laboratory house mice against repeated tick feeding which was unaffected by a concurrent nematode infection.

### Concurrent H. polygyrus infection does not significantly affect Th1 responses to tick-borne B. afzelii infection.

**FIG 6:**
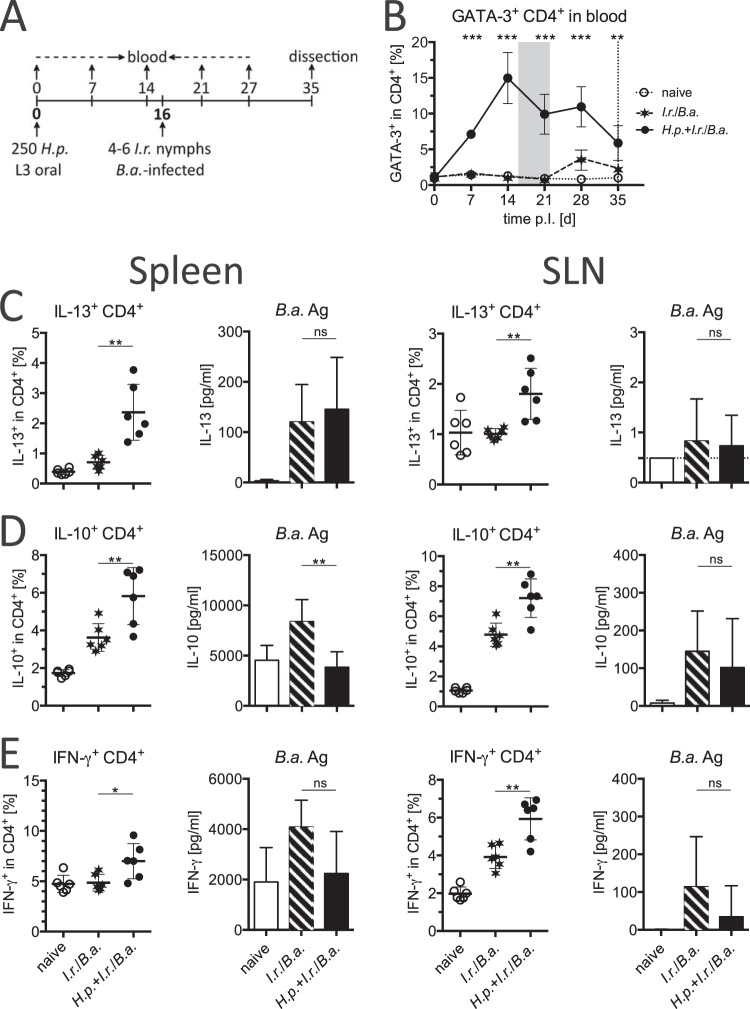
Systemic and local immune responses in mice coinfected with H. polygyrus and I. ricinus-transmitted B. afzelii (*B. a*.). (A) Experimental setup showing the time line (in days) of exposure to parasites and blood sampling. (B) Kinetics of GATA-3^+^ CD4^+^ cell frequencies (mean ± SD) in peripheral blood. Gray shading and the vertical line, period of nymphal tick feeding and the day of dissection, respectively. Asterisks indicate significant differences between the coinfected group and the group infested with I. ricinus only for every time point. (C to E) Production of IL-13 (C), IL-10 (D), and IFN-γ (E) by spleen and SLN CD4^+^ T cells stimulated with PMA-ionomycin and bulk cultures stimulated with B. afzelii antigen. Means and SDs are shown. Symbols represent the intracellular cytokine responses detected in cells from individual mice. Differences between groups were analyzed using unpaired *t* tests. *, *P* < 0.05; **, *P* < 0.01; ***, *P* < 0.001; ns, not significant. Data are for 6 mice per group.

Next, we examined whether increased systemic Th2 responses in mice coinfected with ticks and intestinal nematodes might inhibit Th1 responses against tick-borne spirochetes. Borrelia afzelii-infected I. ricinus nymphs were permitted to feed on mice infected with H. polygyrus and naive controls. As the maximum systemic Th2 response, based on peripheral blood Th2 cell frequencies, occurred in the second week of H. polygyrus infections (Fig. 3C), we chose day 16 after nematode infection for tick attachment (Fig. 6A). High frequencies of GATA-3^+^ Th2 cells in the peripheral blood briefly before and during tick feeding were confirmed (Fig. 6B). Mice were dissected 19 days after I. ricinus infestation, when B. afzelii dissemination in the natural rodent hosts results in maximum infectivity for feeding tick larvae (3). Upon PMA-ionomycin-induced stimulation, spleen cells from mice coinfected with nematodes and ticks again responded with the highest frequencies of CD4^+^ cells producing IL-13, IL-10, and IFN-γ (Fig. 6C to E). A similar response to PMA-ionomycin-induced stimulation was observed for local CD4^+^ T cells derived from SLNs (Fig. 6C to E). To survey antispirochete responses, bulk cultures were stimulated with B. afzelii antigen. Surprisingly, low levels of spirochete-specific IL-13 production by splenocytes but not by SLN cells were detectable in both groups of mice infested with B. afzelii-infected ticks (Fig. 6C). Splenocytes from mice infested only with Borrelia-infected ticks produced high levels of IL-10 in response to spirochete antigen, but this response was significantly suppressed in spleen cells derived from mice coinfected with nematodes and ticks. In contrast, spirochete-specific IL-10 production by local SLN cells was unaffected by the presence of intestinal nematodes (Fig. 6D). Although the highest frequencies of CD4^+^ IFN-γ^+^ cells were detected in the spleens and SLNs of coinfected mice, the B. afzelii antigen-specific responses of splenocytes and SLN cells appeared to be slightly but not significantly lower in mice coinfected with nematodes and ticks, which was also reflected by anti-CD3/anti-CD28 antibody stimulation (Fig. 6D and not shown). Thus, systemic and local Borrelia-specific Th1 reactivity is only mildly affected, if it is affected at all, by a concurrent intestinal nematode infection.

### H. polygyrus infection does not affect the transmission or control of tick-borne B. afzelii infection.

**FIG 7:**
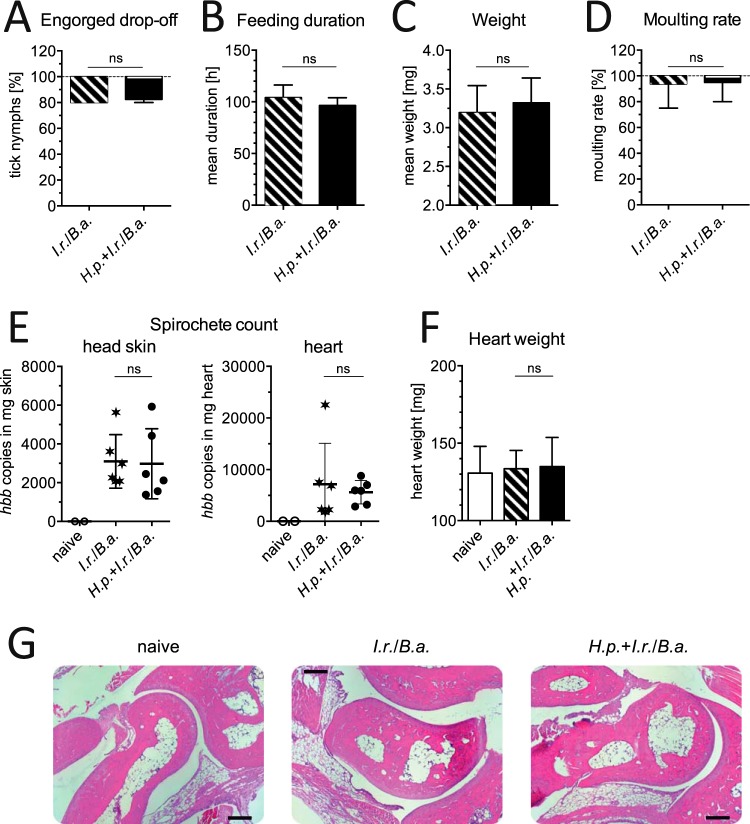
Feeding parameters, spirochete load in mouse tissues, and tibiotarsal joint histology after infection with H. polygyrus and tick-B. afzelii infection. (A and D) The number of engorged nymphs recovered after detachment (A) and rates of molting to adult ticks (D) are depicted as box plots (medians and quartiles, with whiskers representing 95% CIs). The dotted line in panel A depicts the total number of ticks applied to individual mice, and that in panel D depicts the total number of engorged ticks. (B and C) Feeding duration (B) and tick weight after detachment (C) are shown as means ± SDs. (E) Number (mean ± SD) of B. afzelii spirochetes per milligram of head skin (left) and heart (right), measured by absolute quantification of the number of copies of the *hbb* gene via touchdown qPCR. (F) Heart weight of mice (mean ± SD). (G) Representative cross sections from tibiotarsal joints stained with hematoxylin and eosin for histopathological scoring. Bar, 100 μm. Differences between groups were tested using unpaired *t* tests (B, C, E, F) or the Mann-Whitney U test (A, D). ns, not significant. Data are for 6 mice per group.

Because coinfections with intestinal nematodes reduce the control of concomitant bacterial infections (22–24), we examined whether the transmission and dissemination of tick-borne LD spirochetes were affected in nematode-infected hosts. As was observed during primary infestation with larval I. ricinus, H. polygyrus did not affect the feeding success of B. afzelii-infected I. ricinus nymphs (Fig. 7A to D). Nearly all ticks engorged and subsequently molted successfully. We quantified the number of B. afzelii bacteria in the skin of the head, the heart, and tibiotarsal joint by applying a newly established touchdown real-time PCR assay quantifying spirochetes in relation to organ weight. All mice had acquired B. afzelii from the infected nymphal I. ricinus ticks. They harbored about 3,000 spirochetes per mg head skin and about 6,400 spirochetes per mg heart, while the spirochete numbers in the tibiotarsal joints were under the detection limit (Fig. 7E). Spirochete numbers were independent of the presence of H. polygyrus infections. All mice were free of clinical signs of Lyme disease, such as carditis or arthritis. The weight of the hearts of infected mice was comparable to that of the hearts of naive mice (Fig. 7F), and the low numbers of spirochetes that had disseminated to the joints did not lead to signs of pathological inflammation (Fig. 7G). Furthermore, food and water intake, mobility, and body weight were unaltered in B. afzelii-infected mice (data not shown). Taken together, we demonstrate that coinfection with an intestinal nematode fails to facilitate the proliferation and systemic dissemination of tick-borne Lyme disease spirochetes in house mice under experimentally controlled conditions.

## DISCUSSION

Small rodents serve as reservoir hosts for several tick-borne pathogens in Europe, and the I. ricinus tick is the vector for the agents of Lyme disease. Numerous mice and voles inhabit urban areas and thus constitute a considerable source for tick-borne pathogens in close proximity to humans. Hosts serving as competent reservoirs for tick-borne pathogens must permit repeated tick feeding, must be susceptible to the pathogen, and must become and remain infectious for feeding ticks for prolonged periods. Acquired immunity to repeated tick infestations, observed in some artificial hosts, concomitantly reduces the susceptibility to tick-borne pathogens and thus curtails the pathogens' transmission to a new vector (44–46). Currently, it is not known whether and to what extent coinfections affect the susceptibility of rodents to tick feeding and the competence of rodents as a reservoir for tick-borne pathogens. As nematode coinfections are highly prevalent in wild mice and the worm burdens can reach high levels (9–11), they might influence the success of pathogen transmission by repeated tick feeding and the immune control of tick-borne pathogens, such as B. afzelii. Thus, coinfections of rodents with macroparasites may increase the efficiency of transmission of tick-borne pathogens and thereby affect public health. In this study, we present epidemiological data derived from wild mice showing that coinfections with ticks and nematodes are very frequent and combine them with immunological data and parameters related to the competence of the rodent as a reservoir for LD spirochetes obtained from a defined experimental laboratory system. This system was chosen by consideration of the fact that factors likely to affect the surveyed parameters, such as preexposure to tick infestations and LD spirochetes, the time since spirochete infection, and the number of bites by infected ticks, were unknown for rodents trapped in the wild.

We analyzed wild mice captured in Berlin for the occurrence of a Heligmosomoides sp. and I. ricinus and found that both are frequent parasites in wild mice from urban and periurban sites in Berlin; i.e., 41.3% of tick-infested Apodemus mice were coinfected by the nematode. This is in line with the findings of a previous Italian field study reporting that 68% of tick-infested yellow-necked mice were coinfected with H. polygyrus (47). A regression analysis revealed that rodent species and the trapping location mainly affect the number of ticks on the rodents. Only a trend for a negative influence of the number of Heligmosomoides organisms on the tick count was observed, although it did not reach statistical significance. Ferrari et al. (47) reported that besides the trapping location and different host factors, H. polygyrus had a negative influence on the number of I. ricinus ticks infesting wild A. flavicollis mice. These statistical results suggest that the nematode infection may influence the tick infestation, but validation by use of an experimental infection under defined laboratory conditions was lacking. Hence, we examined experimentally whether concurrent nematode infections influence the development of (i) antitick immunity and (ii) the control of the tick-borne pathogen B. afzelii.

Antitick immunity is associated with the development of Th2 immune responses and is mainly accomplished by basophils, mast cells, and eosinophils, which are Th2-associated innate effector cells (13, 42, 48). In our experimental model, C57BL/6 mice acquired partial immunity against repeated tick infestations, detected as a significantly reduced number of larval ticks and a significantly reduced engorgement weight of the larval ticks after the third infestation. A reduction in feeding time during a third tick infestation, as previously documented in BALB/c mice (16), was also evident in our study but may not reliably reflect the immune status of the infested host (49). Our observations during repeated larval infestations are in contrast to those in a study in which several laboratory inbred mouse strains, including C57BL/6 mice, did not develop protective immunity in response to repeated feeding of nymphal I. ricinus ticks (13). It is known that the pattern of tick antigenic molecules recognized by the tick hosts is dependent on the tick life stage (50); therefore, it is possible that the larval Ixodes stage is more immunogenic or more susceptible to discrete immune defense mechanisms than the nymphal stage.

In our experimental coinfections of laboratory mice with nematodes and ticks, the systemic Th2 immune responses against the distinct macroparasites resulted in the generation of extraordinarily high levels of Th2 cells in the peripheral blood and spleens, and these peaked during the second tick infestation and simultaneous acute nematode infection. This, however, did not affect the anti-tick Th2 responses measured locally in skin-draining lymph nodes after repeated infestations with larval ticks. Consequently, the success of larval tick feeding was unaffected by prepatent, acute, and chronic nematode coinfection, and partial immunity to tick feeding developed independently of the concurrent nematode infection. In addition, infestation with infected nymphal ticks was unaffected. Taken together, we demonstrate that a concurrent H. polygyrus infection does not reduce the susceptibility of mice to tick feeding.

A previous study revealed a negative correlation between H. polygyrus infection (the number of eggs per gram in feces) and tick numbers on wild yellow-necked mice and showed that tick infestation was increased when mice were dewormed, released, and recaptured compared to the level of infestation for the nontreated controls (47). Competition for space and food could not explain that correlation between these two macroparasites, and the influence of factors such as sex and host sex-related behavior, age, breeding condition, and habitat was at least partly ruled out, as the sites where the treated and control mice were trapped were in close proximity and similar parasite abundances were observed on the mice *a priori* (47). The intensity of grooming behavior leading to reduced tick infestation and increased ingestion of nematode larvae from the fur could also explain the negative correlation but not the increased level of tick infestation after deworming in the study of Ferrari et al. (47). Our findings suggest that the possibility of an immunologic basis for this negative correlation or the release of toxic products by one parasite affecting the other, which was suggested by the authors of the previous study (47), can also be excluded.

Several reasons may account for the differences observed between the mice obtained from the wild and those raised in the laboratory. First, the antitick immune responses and susceptibility to coinfection-induced immune changes observed in the Mus musculus mice used in our experimental study may differ from those observed in other mouse species, such as A. flavicollis and A. sylvaticus. Second, recent comparative immunological analyses show that SPF laboratory mice and those obtained from the wild differ not only in their expression of immune functions but also in specific and generalized immune reactivity. C57BL/6 mice raised under SPF conditions activate natural killer cells less readily than wild M. musculus mice (51), which also display a stronger general status of immune cell activation and higher antibody titers and avidities (52). Thus, coinfections with nematodes may differentially affect the success of tick feeding and antitick immunity in wild mice with a highly activated immune system. Third, sex-related differences in the immune reactivity of the host to many parasite species have been described (47, 53, 54). We restricted our experimental coinfection studies to female mice, but factors such as host sex, breeding condition, nutritional status, and population density may clearly alter immune reactivity to single infections and coinfections with parasites.

Infections with intestinal nematodes affect immune responses to unrelated pathogens, such as bacteria and protozoa (22–28). This results from efficient immune modulation by parasitic worms and the counterregulation of Th1 and associated cytotoxic T cell responses (25, 27), as well as modulated innate effector cell functions during strongly biased Th2 responses (22, 55). Immune control of LD spirochetes depends on the development of Th1 responses in mice and humans (56–59). In the present study, mice coinfected with H. polygyrus and I. ricinus were only slightly inhibited in their mounting of a Th1 response to B. afzelii, as seen by a trend of reduced IFN-γ production by local and systemic T cells in response to the spirochete antigen. Surprisingly, the general capacity for IFN-γ production, as assessed after unspecific CD4^+^ T cell activation, was significantly elevated even in mice that had been coinfected/infested with nematodes and ticks, further arguing against a counterregulation of Th1 reactivity in the face of two macroparasites coinducing strong Th2 responses. We also assessed whether a concurrent nematode infection increased local and systemic spirochete-specific IL-10 levels; however, local responses were not altered and the Borrelia-specific IL-10 production was significantly suppressed in the splenocytes of mice coinfected with nematodes. In line with these data, the presence of an acute worm infection at the peak of systemic Th2 cell reactions did not affect either the susceptibility of mice to B. afzelii or spirochetal proliferation or dissemination to different organs. Because two of the three essential components of reservoir competence, susceptibility to repeated tick feeding and susceptibility to the vector-borne pathogen, were not influenced, nor were spirochetal numbers modified by coinfection with H. polygyrus, we speculate that the competence of mice as a reservoir for LD spirochetes is not enhanced by the presence of enteric nematodes. Hence, this study indicates that the strong systemic Th2 responses in house mice coinfected with nematodes and ticks fail to affect the success of tick feeding and the control of the causative agent of Lyme disease.

## Supplementary Material

Supplemental material

## References

[1] Stanek G 2009 Pandora's box: pathogens in Ixodes ricinus ticks in Central Europe. Wien Klin Wochenschr 121:673–683. doi:10.1007/s00508-009-1281-9. 19998007

[2] Matuschka FR, Fischer P, Musgrave K, Richter D, Spielman A 1991 Hosts on which nymphal Ixodes ricinus most abundantly feed. Am J Trop Med Hyg 44:1000–1007. 10.4269/ajtmh.1991.44.100 1996733

[3] Richter D, Klug B, Spielman A, Matuschka FR 2004 Adaptation of diverse Lyme disease spirochetes in a natural rodent reservoir host. Infect Immun 72:2442–2444. doi:10.1128/IAI.72.4.2442-2444.2004. 15039378 PMC375191

[4] Matuschka FR, Fischer P, Heiler M, Blümcke S, Spielman A 1992 Stage-associated risk of transmission of the Lyme disease spirochete by European Ixodes ticks. Parasitol Res 78:695–698. doi:10.1007/BF00931523. 1480608

[5] Kurtenbach K, Peacey M, Rijpkema SG, Hoodless AN, Nuttall PA, Randolph SE 1998 Differential transmission of the genospecies of Borrelia burgdorferi sensu lato by game birds and small rodents in England. Appl Environ Microbiol 64:1169–1174. 9546150 10.1128/aem.64.4.1169-1174.1998 PMC106125

[6] Humair PF, Peter O, Wallich R, Gern L 1995 Strain variation of Lyme disease spirochetes isolated from Ixodes ricinus ticks and rodents collected in two endemic areas in Switzerland. J Med Entomol 32:433–438. doi:10.1093/jmedent/32.4.433. 7650703

[7] Kurtenbach K, De Michelis S, Etti S, Schäfer SM, Sewell HS, Brade V, Kraiczy P 2002 Host association of Borrelia burgdorferi sensu lato—the key role of host complement. Trends Microbiol 10:74–79. doi:10.1016/S0966-842X(01)02298-3. 11827808

[8] Radolf JD, Caimano MJ, Stevenson B, Hu LT 2012 Of ticks, mice and men: understanding the dual-host lifestyle of Lyme disease spirochaetes. Nat Rev Microbiol 10:87–99. doi:10.1038/nrmicro2714. 22230951 PMC3313462

[9] Klimpel S, Forster M, Schmahl G 2007 Parasites of two abundant sympatric rodent species in relation to host phylogeny and ecology. Parasitol Res 100:867–875. doi:10.1007/s00436-006-0368-8. 17120043

[10] Stammer HJ 1955 Die Parasiten deutscher Kleinsäuger. Verhandlungen Dtsch Zool Ges Erlangen 16:362–390.

[11] Schmidt R 1961 Untersuchungen über die Entoparasitenfauna des Magen-Darmtraktes und der Leibeshöhle von Muriden (Rodentia) der Umgebung Halles unter besonderer Berücksichtigung der Cestoden und Nematoden. Wiss Z Martin-Luther Univ Halle-Wittenberg Math-Nat 11:457–470.

[12] Christe M, Rutti B, Brossard M 1998 Susceptibility of BALB/c mice to nymphs and larvae of Ixodes ricinus after modulation of IgE production with anti-interleukin-4 or anti-interferon-gamma monoclonal antibodies. Parasitol Res 84:388–393. doi:10.1007/s004360050415. 9610636

[13] Christe M, Rutti B, Brossard M 1999 Influence of the genetic background and parasite load of mice on the immune response developed against nymphs of Ixodes ricinus. Parasitol Res 85:557–561. doi:10.1007/s004360050595. 10382605

[14] Mbow ML, Christe M, Rutti B, Brossard M 1994 Absence of acquired resistance to nymphal Ixodes ricinus ticks in BALB/c mice developing cutaneous reactions. J Parasitol 80:81–87. doi:10.2307/3283349. 8308662

[15] Dizij A, Kurtenbach K 1995 Clethrionomys glareolus, but not Apodemus flavicollis, acquires resistance to Ixodes ricinus L., the main European vector of Borrelia burgdorferi. Parasite Immunol 17:177–183. doi:10.1111/j.1365-3024.1995.tb00887.x. 7624158

[16] Borský I, Hermánek J, Uhlír J, Dusbábek F 1994 Humoral and cellular immune response of BALB/c mice to repeated infestations with Ixodes ricinus nymphs. Int J Parasitol 24:127–132. doi:10.1016/0020-7519(94)90066-3. 8021100

[17] Zeidner N, Mbow ML, Dolan M, Massung R, Baca E, Piesman J 1997 Effects of Ixodes scapularis and Borrelia burgdorferi on modulation of the host immune response: induction of a TH2 cytokine response in Lyme disease-susceptible (C3H/HeJ) mice but not in disease-resistant (BALB/c) mice. Infect Immun 65:3100–3106. 9234760 10.1128/iai.65.8.3100-3106.1997 PMC175437

[18] Zeidner N, Dreitz M, Belasco D, Fish D 1996 Suppression of acute Ixodes scapularis-induced Borrelia burgdorferi infection using tumor necrosis factor-alpha, interleukin-2, and interferon-gamma. J Infect Dis 173:187–195. doi:10.1093/infdis/173.1.187. 8537658

[19] Zeidner NS, Schneider BS, Rutherford JS, Dolan MC 2008 Suppression of Th2 cytokines reduces tick-transmitted Borrelia burgdorferi load in mice. J Parasitol 94:767–769. doi:10.1645/GE-1416.1. 18605798

[20] Mohrs K, Harris DP, Lund FE, Mohrs M 2005 Systemic dissemination and persistence of Th2 and type 2 cells in response to infection with a strictly enteric nematode parasite. J Immunol 175:5306–5313. doi:10.4049/jimmunol.175.8.5306. 16210636

[21] Maizels RM, Hewitson JP, Murray J, Harcus YM, Dayer B, Filbey KJ, Grainger JR, McSorley HJ, Reynolds LA, Smith KA 2012 Immune modulation and modulators in Heligmosomoides polygyrus infection. Exp Parasitol 132:76–89. doi:10.1016/j.exppara.2011.08.011. 21875581 PMC6485391

[22] Chen CC, Louie S, McCormick B, Walker WA, Shi HN 2005 Concurrent infection with an intestinal helminth parasite impairs host resistance to enteric Citrobacter rodentium and enhances Citrobacter-induced colitis in mice. Infect Immun 73:5468–5481. doi:10.1128/IAI.73.9.5468-5481.2005. 16113263 PMC1231118

[23] Chen CC, Louie S, McCormick BA, Walker WA, Shi HN 2006 Helminth-primed dendritic cells alter the host response to enteric bacterial infection. J Immunol 176:472–483. doi:10.4049/jimmunol.176.1.472. 16365440 PMC4144328

[24] Su CW, Cao Y, Zhang M, Kaplan J, Su L, Fu Y, Walker WA, Xavier R, Cherayil BJ, Shi HN 2012 Helminth infection impairs autophagy-mediated killing of bacterial enteropathogens by macrophages. J Immunol 189:1459–1466. doi:10.4049/jimmunol.1200484. 22732589 PMC3423331

[25] Tetsutani K, Ishiwata K, Ishida H, Tu L, Torii M, Hamano S, Himeno K, Hisaeda H 2009 Concurrent infection with Heligmosomoides polygyrus suppresses anti-Plasmodium yoelii protection partially by induction of CD4(+)CD25(+)Foxp3(+) Treg in mice. Eur J Immunol 39:2822–2830. doi:10.1002/eji.200939433. 19728313

[26] Helmby H 2009 Gastrointestinal nematode infection exacerbates malaria-induced liver pathology. J Immunol 182:5663–5671. doi:10.4049/jimmunol.0803790. 19380813 PMC2796717

[27] Khan IA, Hakak R, Eberle K, Sayles P, Weiss LM, Urban JF Jr 2008 Coinfection with Heligmosomoides polygyrus fails to establish CD8^+^ T-cell immunity against Toxoplasma gondii. Infect Immun 76:1305–1313. doi:10.1128/IAI.01236-07. 18195022 PMC2258819

[28] Rausch S, Held J, Stange J, Lendner M, Hepworth MR, Klotz C, Lucius R, Pogonka T, Hartmann S 2010 A matter of timing: early, not chronic phase intestinal nematode infection restrains control of a concurrent enteric protozoan infection. Eur J Immunol 40:2804–2815. doi:10.1002/eji.201040306. 20809519

[29] Anderson RC, Chabaud AG, Willmott S 2009 Keys to the nematode parasites of vertebrates: archival volume. CABI Publishing, Wallingford, Oxfordshire, United Kingdom.

[30] Morel P, Perez C 1978 Morphologie des stades préimaginaux des Ixodida s. str. d'Europe occidentale. V. Les larves des Ixodes s. str. Acarologia 19:395–405.

[31] Morel P, Perez C 1978 Morphologie des stades préimaginaux des Ixodida s. str. d'Europe occidentale. VI. Les nymphes des Ixodes s. str. Acarologia 19:579–586.

[32] Marquez FJ, Morel PC, Guiguen C, Beaucournu JC 1992 Clé dichotomique des Ixodidae d'Europe. I. Les larves du genre Ixodes. Acarologia 33:325–330.

[33] Schreiber C, Krücken J, Beck S, Maaz D, Pachnicke S, Krieger K, Gross M, Kohn B, von Samson-Himmelstjerna G 2014 Pathogens in ticks collected from dogs in Berlin/Brandenburg, Germany. Parasit Vectors 7:535. doi:10.1186/s13071-014-0535-1. 25441762 PMC4262381

[34] Krücken J, Schreiber C, Maaz D, Kohn M, Demeler J, Beck S, Schein E, Olias P, Richter D, Matuschka FR, Pachnicke S, Krieger K, Kohn B, von Samson-Himmelstjerna G 2013 A novel high-resolution melt PCR assay discriminates Anaplasma phagocytophilum and “Candidatus Neoehrlichia mikurensis.” J Clin Microbiol 51:1958–1961. doi:10.1128/JCM.00284-13. 23576542 PMC3716091

[35] Peine M, Rausch S, Helmstetter C, Fröhlich A, Hegazy AN, Kühl AA, Grevelding CG, Höfer T, Hartmann S, Löhning M 2013 Stable T-bet(+)GATA-3(+) Th1/Th2 hybrid cells arise in vivo, can develop directly from naive precursors, and limit immunopathologic inflammation. PLoS Biol 11:e1001633. doi:10.1371/journal.pbio.1001633. 23976880 PMC3747991

[36] Portnoï D, Sertour N, Ferquel E, Garnier M, Baranton G, Postic D 2006 A single-run, real-time PCR for detection and identification of Borrelia burgdorferi sensu lato species, based on the hbb gene sequence. FEMS Microbiol Lett 259:35–40. doi:10.1111/j.1574-6968.2006.00249.x. 16684099

[37] Filbey KJ, Grainger JR, Smith KA, Boon L, van Rooijen N, Harcus Y, Jenkins S, Hewitson JP, Maizels RM 2014 Innate and adaptive type 2 immune cell responses in genetically controlled resistance to intestinal helminth infection. Immunol Cell Biol 92:436–448. doi:10.1038/icb.2013.109. 24492801 PMC4038150

[38] Ma Y, Seiler KP, Eichwald EJ, Weis JH, Teuscher C, Weis JJ 1998 Distinct characteristics of resistance to Borrelia burgdorferi-induced arthritis in C57BL/6N mice. Infect Immun 66:161–168. 9423853 10.1128/iai.66.1.161-168.1998 PMC107872

[39] Rausch S, Huehn J, Loddenkemper C, Hepworth MR, Klotz C, Sparwasser T, Hamann A, Lucius R, Hartmann S 2009 Establishment of nematode infection despite increased Th2 responses and immunopathology after selective depletion of Foxp3^+^ cells. Eur J Immunol 39:3066–3077. doi:10.1002/eji.200939644. 19750483

[40] Rausch S, Huehn J, Kirchhoff D, Rzepecka J, Schnoeller C, Pillai S, Loddenkemper C, Scheffold A, Hamann A, Lucius R, Hartmann S 2008 Functional analysis of effector and regulatory T cells in a parasitic nematode infection. Infect Immun 76:1908–1919. doi:10.1128/IAI.01233-07. 18316386 PMC2346705

[41] Finney CA, Taylor MD, Wilson MS, Maizels RM 2007 Expansion and activation of CD4(+)CD25(+) regulatory T cells in Heligmosomoides polygyrus infection. Eur J Immunol 37:1874–1886. doi:10.1002/eji.200636751. 17563918 PMC2699425

[42] Wada T, Ishiwata K, Koseki H, Ishikura T, Ugajin T, Ohnuma N, Obata K, Ishikawa R, Yoshikawa S, Mukai K, Kawano Y, Minegishi Y, Yokozeki H, Watanabe N, Karasuyama H 2010 Selective ablation of basophils in mice reveals their nonredundant role in acquired immunity against ticks. J Clin Invest 120:2867–2875. doi:10.1172/JCI42680. 20664169 PMC2912199

[43] Matsuda H, Fukui K, Kiso Y, Kitamura Y 1985 Inability of genetically mast cell-deficient W/Wv mice to acquire resistance against larval Haemaphysalis longicornis ticks. J Parasitol 71:443–448. doi:10.2307/3281535. 3897501

[44] Titus RG, Bishop JV, Mejia JS 2006 The immunomodulatory factors of arthropod saliva and the potential for these factors to serve as vaccine targets to prevent pathogen transmission. Parasite Immunol 28:131–141. 16542315 10.1111/j.1365-3024.2006.00807.x

[45] Nazario S, Das S, de Silva AM, Deponte K, Marcantonio N, Anderson JF, Fish D, Fikrig E, Kantor FS 1998 Prevention of Borrelia burgdorferi transmission in guinea pigs by tick immunity. Am J Trop Med Hyg 58:780–785. 9660463 10.4269/ajtmh.1998.58.780

[46] Jones LD, Nuttall PA 1990 The effect of host resistance to tick infestation on the transmission of Thogoto virus by ticks. J Gen Virol 71:1039–1043. doi:10.1099/0022-1317-71-5-1039. 2345364

[47] Ferrari N, Cattadori IM, Rizzoli A, Hudson PJ 2009 Heligmosomoides polygyrus reduces infestation of Ixodes ricinus in free-living yellow-necked mice, Apodemus flavicollis. Parasitology 136:305–316. doi:10.1017/S0031182008005404. 19154651

[48] Mejri N, Brossard M 2007 Splenic dendritic cells pulsed with Ixodes ricinus tick saliva prime naive CD4^+^ T to induce Th2 cell differentiation *in vitro* and *in vivo*. Int Immunol 19:535–543. doi:10.1093/intimm/dxm019. 17344202

[49] Fujisaki K 1978 Development of acquired resistance precipitating antibody in rabbits experimentally infested with females of Haemaphysalis longicornis (Ixodoidea: Ixodidae). Natl Inst Anim Health Q (Tokyo) 18:27–38. 77478

[50] Lawrie CH, Nuttall PA 2001 Antigenic profile of Ixodes ricinus: effect of developmental stage, feeding time and the response of different host species. Parasite Immunol 23:549–556. doi:10.1046/j.1365-3024.2001.00412.x. 11696166

[51] Boysen P, Eide DM, Storset AK 2011 Natural killer cells in free-living Mus musculus have a primed phenotype. Mol Ecol 20:5103–5110. doi:10.1111/j.1365-294X.2011.05269.x. 21895821

[52] Abolins SR, Pocock MJ, Hafalla JC, Riley EM, Viney ME 2011 Measures of immune function of wild mice, Mus musculus. Mol Ecol 20:881–892. doi:10.1111/j.1365-294X.2010.04910.x. 21073587

[53] Hepworth MR, Hardman MJ, Grencis RK 2010 The role of sex hormones in the development of Th2 immunity in a gender-biased model of Trichuris muris infection. Eur J Immunol 40:406–416. doi:10.1002/eji.200939589. 19950176 PMC3549561

[54] Klein SL 2004 Hormonal and immunological mechanisms mediating sex differences in parasite infection. Parasite Immunol 26:247–264. doi:10.1111/j.0141-9838.2004.00710.x. 15541029

[55] Weng M, Huntley D, Huang IF, Foye-Jackson O, Wang L, Sarkissian A, Zhou Q, Walker WA, Cherayil BJ, Shi HN 2007 Alternatively activated macrophages in intestinal helminth infection: effects on concurrent bacterial colitis. J Immunol 179:4721–4731. doi:10.4049/jimmunol.179.7.4721. 17878371 PMC3208515

[56] Sjöwall J, Fryland L, Nordberg M, Sjögren F, Garpmo U, Jansson C, Carlsson SA, Bergström S, Ernerudh J, Nyman D, Forsberg P, Ekerfelt C 2011 Decreased Th1-type inflammatory cytokine expression in the skin is associated with persisting symptoms after treatment of erythema migrans. PLoS One 6:e18220. doi:10.1371/journal.pone.0018220. 21483819 PMC3069060

[57] Kang I, Barthold SW, Persing DH, Bockenstedt LK 1997 T-helper-cell cytokines in the early evolution of murine Lyme arthritis. Infect Immun 65:3107–3111. 9234761 10.1128/iai.65.8.3107-3111.1997 PMC175438

[58] Forsberg P, Ernerudh J, Ekerfelt C, Roberg M, Vrethem M, Bergström S 1995 The outer surface proteins of Lyme disease borrelia spirochetes stimulate T cells to secrete interferon-gamma (IFN-gamma): diagnostic and pathogenic implications. Clin Exp Immunol 101:453–460. 7664493 10.1111/j.1365-2249.1995.tb03134.x PMC1553228

[59] Widhe M, Jarefors S, Ekerfelt C, Vrethem M, Bergstrom S, Forsberg P, Ernerudh J 2004 Borrelia-specific interferon-gamma and interleukin-4 secretion in cerebrospinal fluid and blood during Lyme borreliosis in humans: association with clinical outcome. J Infect Dis 189:1881–1891. doi:10.1086/382893. 15122525

