# Supplementary material for: Susceptibility to Ticks and Lyme Disease Spirochetes Is Not Affected in Mice Coinfected with Nematodes
Source: Infect Immun. 2016 Apr 22;84(5):1274–86. doi: 10.1128/IAI.01309-15 (PMC4862734; doi:10.1128/IAI.01309-15)
Supplement: Supplemental material [file supp_84_5_1274__index.html]

Supplemental material 

# Susceptibility to Ticks and Lyme Disease Spirochetes Is Not Affected in Mice Coinfected with Nematodes

## Supplemental material

- Supplemental file 1 -

  Fig. S1. Histological analysis of local Th2-associated innate effector cells in skin.

  PDF, 184K
