# Supplementary material for: Susceptibility to Ticks and Lyme Disease Spirochetes Is Not Affected in Mice Coinfected with Nematodes
Source: Infect Immun. 2016 Apr 22;84(5):1274–86. doi: 10.1128/IAI.01309-15 (PMC4862734; doi:10.1128/IAI.01309-15)
Supplement: Supplemental material [file IAI.01309-15_zii999091677so1.pdf]

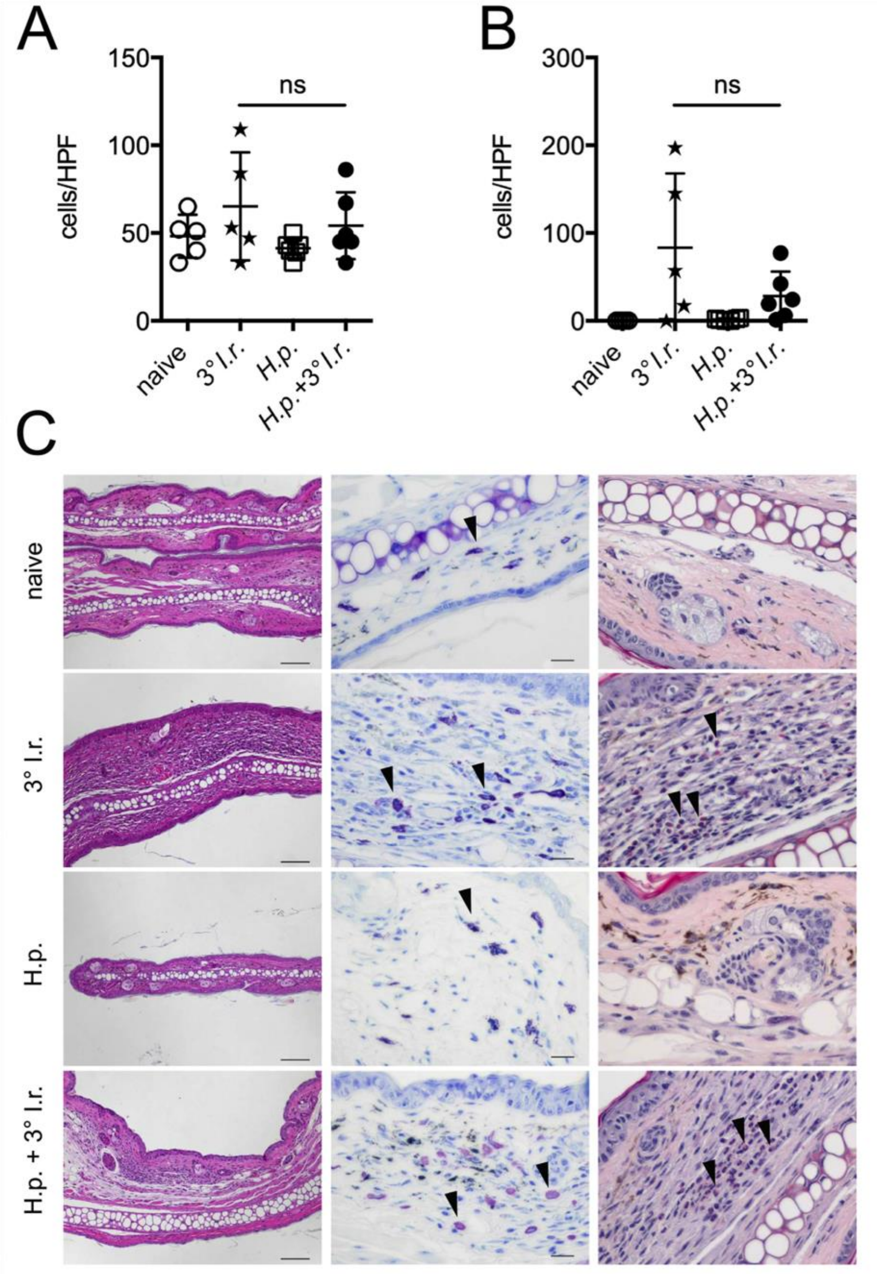

Figure S1. Histological analysis of local Th2-associated innate effector cells in skin. Number of (A) mast cells and (B) eosinophils as determined in histological ear skin sections (mean ±SD). ns: not significant. N=5-6 mice/group. Differences between groups were analysed using unpaired t-test (A) or Mann-Whitney-U-test (B). (C) Representative examples of skin sections stained with H&E (left column, scale bar 100µm), toluidine blue for mast cell detection (mid column, scale bar 20µm) and Sirius red for detection of eosinophils (right column, scale bar 20µm). Arrowheads depict mast cells and eosinophils, respectively.
